# Mammographic density and ageing: A collaborative pooled analysis of cross-sectional data from 22 countries worldwide

**DOI:** 10.1371/journal.pmed.1002335

**Published:** 2017-06-30

**Authors:** Anya Burton, Gertraud Maskarinec, Beatriz Perez-Gomez, Celine Vachon, Hui Miao, Martín Lajous, Ruy López-Ridaura, Megan Rice, Ana Pereira, Maria Luisa Garmendia, Rulla M. Tamimi, Kimberly Bertrand, Ava Kwong, Giske Ursin, Eunjung Lee, Samera A. Qureshi, Huiyan Ma, Sarah Vinnicombe, Sue Moss, Steve Allen, Rose Ndumia, Sudhir Vinayak, Soo-Hwang Teo, Shivaani Mariapun, Farhana Fadzli, Beata Peplonska, Agnieszka Bukowska, Chisato Nagata, Jennifer Stone, John Hopper, Graham Giles, Vahit Ozmen, Mustafa Erkin Aribal, Joachim Schüz, Carla H. Van Gils, Johanna O. P. Wanders, Reza Sirous, Mehri Sirous, John Hipwell, Jisun Kim, Jong Won Lee, Caroline Dickens, Mikael Hartman, Kee-Seng Chia, Christopher Scott, Anna M. Chiarelli, Linda Linton, Marina Pollan, Anath Arzee Flugelman, Dorria Salem, Rasha Kamal, Norman Boyd, Isabel dos-Santos-Silva, Valerie McCormack

**Affiliations:** 1 Section of Environment and Radiation, International Agency for Research on Cancer, Lyon, France; 2 University of Hawaii Cancer Center, Honolulu, Hawaii, United States of America; 3 Instituto de Salud Carlos III, Madrid, Spain; 4 CIBERESP, Madrid, Spain; 5 Department of Health Sciences Research, Mayo Clinic, Rochester, Minnesota, United States of America; 6 Saw Swee Hock School of Public Health, National University of Singapore, Singapore; 7 Instituto Nacional de Salud Pública, Cuernavaca, Mexico; 8 Massachusetts General Hospital, Harvard Medical School, Boston, Massachusetts, United States of America; 9 Instituto de Nutrición y Tecnología de los Alimentos, Universidad de Chile, Santiago, Chile; 10 Brigham and Women’s Hospital, Harvard Medical School, Boston, Massachusetts, United States of America; 11 Slone Epidemiology Center, Boston University, Boston, Massachusetts, United States of America; 12 Division of Breast Surgery, Li Ka Shing Faculty of Medicine, University of Hong Kong, Hong Kong, China; 13 Department of Surgery and Cancer Genetics Center, Hong Kong Sanatorium and Hospital, Hong Kong, China; 14 Hong Kong Hereditary Breast Cancer Family Registry, Hong Kong, China; 15 Cancer Registry of Norway, Oslo, Norway; 16 Department of Nutrition, Institute of Basic Medical Sciences, University of Oslo, Oslo, Norway; 17 Department of Preventive Medicine, Keck School of Medicine, University of Southern California, Los Angeles, United States of America; 18 Norwegian Centre for Migrant and Minority Health (NAKMI), Oslo, Norway; 19 Department of Population Sciences, City of Hope National Medical Center, Duarte, California, United States of America; 20 Division of Cancer Research, Ninewells Hospital and Medical School, Dundee, United Kingdom; 21 Wolfson Institute of Preventive Medicine, Queen Mary University of London, London, United Kingdom; 22 Department of Diagnostic Radiology, Royal Marsden NHS Foundation Trust, London, United Kingdom; 23 Aga Khan University Hospital, Nairobi, Kenya; 24 Breast Cancer Research Group, University of Malaya Medical Centre, University of Malaya, Kuala Lumpur, Malaysia; 25 Cancer Research Malaysia, Subang Jaya, Malaysia; 26 Breast Cancer Research Unit, Faculty of Medicine, University of Malaya Cancer Research Institute, University of Malaya, Kuala Lumpur, Malaysia; 27 Biomedical Imaging Department, University of Malaya Medical Centre, University of Malaya, Kuala Lumpur, Malaysia; 28 Nofer Institute of Occupational Medicine, Łódź, Poland; 29 Department of Epidemiology & Preventive Medicine, Graduate School of Medicine, Gifu University, Gifu, Japan; 30 Centre for Genetic Origins of Health and Disease, University of Western Australia, Crawley, Western Australia, Australia; 31 Centre for Epidemiology and Biostatistics, Melbourne School of Population and Global Health, University of Melbourne, Melbourne, Victoria, Australia; 32 Cancer Epidemiology Centre, Cancer Council Victoria, Melbourne, Victoria, Australia; 33 Faculty of Medicine, Istanbul University, Istanbul, Turkey; 34 Department of Radiology, School of Medicine, Marmara University, Istanbul, Turkey; 35 Julius Center for Health Sciences and Primary Care, University Medical Center Utrecht, Utrecht, The Netherlands; 36 Isfahan University of Medical Sciences, Isfahan, Iran; 37 Radiology Department, Isfahan University of Medical Sciences, Isfahan, Iran; 38 Centre for Medical Image Computing, University College London, London, United Kingdom; 39 Asan Medical Center, Seoul, Republic of Korea; 40 Department of Internal Medicine, Faculty of Health Sciences, University of the Witwatersrand, Johannesburg, South Africa; 41 Department of Surgery, Yong Loo Lin School of Medicine, Singapore; 42 Graduate School for Integrative Sciences and Engineering, National University of Singapore, Singapore; 43 Ontario Breast Screening Program, Cancer Care Ontario, Toronto, Ontario, Canada; 44 Princess Margaret Cancer Centre, Toronto, Ontario, Canada; 45 National Cancer Control Center, Lady Davis Carmel Medical Center, Faculty of Medicine, Technion–Israel Institute of Technology, Haifa, Israel; 46 Woman Imaging Unit, Radiodiagnosis Department, Kasr El Aini, Cairo University Hospitals, Cairo, Egypt; 47 Department of Non-communicable Disease Epidemiology, London School of Hygiene & Tropical Medicine, London, United Kingdom; Fred Hutchinson Cancer Research Center, UNITED STATES

## Abstract

**Background:**

Mammographic density (MD) is one of the strongest breast cancer risk factors. Its age-related characteristics have been studied in women in western countries, but whether these associations apply to women worldwide is not known.

**Methods and findings:**

We examined cross-sectional differences in MD by age and menopausal status in over 11,000 breast-cancer-free women aged 35–85 years, from 40 ethnicity- and location-specific population groups across 22 countries in the International Consortium on Mammographic Density (ICMD). MD was read centrally using a quantitative method (Cumulus) and its square-root metrics were analysed using meta-analysis of group-level estimates and linear regression models of pooled data, adjusted for body mass index, reproductive factors, mammogram view, image type, and reader. In all, 4,534 women were premenopausal, and 6,481 postmenopausal, at the time of mammography. A large age-adjusted difference in percent MD (PD) between post- and premenopausal women was apparent (–0.46 cm [95% CI: −0.53, −0.39]) and appeared greater in women with lower breast cancer risk profiles; variation across population groups due to heterogeneity (*I*^2^) was 16.5%. Among premenopausal women, the √PD difference per 10-year increase in age was −0.24 cm (95% CI: −0.34, −0.14; *I*^2^ = 30%), reflecting a compositional change (lower dense area and higher non-dense area, with no difference in breast area). In postmenopausal women, the corresponding difference in √PD (−0.38 cm [95% CI: −0.44, −0.33]; I^2^ = 30%) was additionally driven by increasing breast area. The study is limited by different mammography systems and its cross-sectional rather than longitudinal nature.

**Conclusions:**

Declines in MD with increasing age are present premenopausally, continue postmenopausally, and are most pronounced over the menopausal transition. These effects were highly consistent across diverse groups of women worldwide, suggesting that they result from an intrinsic biological, likely hormonal, mechanism common to women. If cumulative breast density is a key determinant of breast cancer risk, younger ages may be the more critical periods for lifestyle modifications aimed at breast density and breast cancer risk reduction.

## Abstract 2

Valerie McCormack and colleagues reveal reduced breast density with increased age in women from different ethnic groups; however increased breast density in young women is a cancer risk.

## Author summary

**Why was this study done?:**

- Breast density, a measure of the amount of dense to fatty tissue in the breast, is a strong marker of breast cancer risk.
- Breast density determinants have been studied in high-income countries, but not in women worldwide.

**What did the researchers do and find?:**

- We studied how breast density differed by age and menopausal status in diverse groups of women from 22 countries worldwide.
- For over 11,000 women, mammograms were read centrally and risk factor data harmonised.
- We found that, regardless of the country and ethnic group of women, breast density was much lower in postmenopausal than in premenopausal women of the same age.
- Additionally, as a relative proportion of breast area, breast density was lower in older women both among premenopausal and postmenopausal women. Among premenopausal women, breast density changed with age without an increase in breast area, but among postmenopausal women, the latter also increased.

**What do these findings mean?:**

- That this change in breast density with age is seen internationally suggests that the change is due to a universal biological mechanism inherent to all women.
- If cumulative breast density is a key determinant of breast cancer risk, younger ages may be the more critical periods for lifestyle modifications aimed at breast density and breast cancer risk reduction.

## Introduction

Mammographic density (MD), a measure of the amount of radiopaque fibroglandular as opposed to fat tissue in the breast, is amongst the strongest of breast cancer risk factors [1–3]. Parallels have been drawn between life-course trajectories of MD and Pike’s model for the rate of breast tissue ageing [1–4]. The latter model hypothesizes that Clemmesen’s hook (the slowing of the rate of increase of age-specific breast cancer incidence rates after menopause, S1 Fig) is due to a reduction in the rate of breast tissue ageing in postmenopausal women [4–6]. MD also declines during the menopausal transition and with ageing; thus, MD may be a tissue-specific marker of the biological processes underlying the rate of breast tissue ageing and, ultimately, the shape of the breast cancer incidence–age curve [2,7,8]. The nature and drivers of the cumulative MD profile thus become of interest to inform which periods in life MD reductions may be best targeted to.

MD associations with ageing have been examined in longitudinal studies [8–12] and inferred from cross-sectional studies [13–15]. Many found non-linear declines with increasing age [8,10–12], often steepest during the perimenopausal period, and some suggested that MD plateaus by age 65 years [11,12]. The majority of these studies were conducted at screening ages (predominantly 50–70 years) and in countries with high breast cancer incidence rates. However, whilst Clemmesen’s hook has been observed in countries spanning the range of breast cancer incidence rates (as shown for selected international populations in S1 Fig), it is not known how MD changes with age in women from countries beyond westernised populations. Studying whether the MD–age association holds internationally will shed light on whether the association is likely to be driven by an intrinsic biology or is a consequence of, or specific to, westernised lifestyles.

The International Consortium on Mammographic Density (ICMD) is an international pooling consortium of cross-sectional individual-level epidemiologic and MD data on over 11,000 breast-cancer-free women from 22 diverse countries. In this consortium, we examined differences in MD by menopausal status and age across 4 decades of life.

## Methods

Ethics approvals for ICMD were obtained from the International Agency for Research on Cancer (IEC 12–34). Each individual participating study had received local ethical approval at the time of the original conduct of the study and again to contribute to the consortium.

The ICMD methodology and contributing studies are discussed in detail elsewhere [16]. In brief, the consortium pooled individual-level epidemiologic and MD data on 11,755 women without breast cancer from 27 studies (listed in S1 Text) in 22 countries that span low to high breast cancer incidence rates. To enhance heterogeneity, 7 studies contributed multiple ethnic groups, e.g., Chinese, Malay, and Indian women in both Singapore and Malaysia, totalling 40 ethnicity- and location-specific ‘population groups’. From each population group, we sought to include a random selection of 200 pre- and 200 postmenopausal women aged 35 years or older at the time of mammography. These sample sizes were based on calculations to allow, within each population group and menopause stratum, estimation of mean percent MD (PD) within 1% at a 95% confidence level, assuming a stratum-specific standard deviation (SD) of 7% (for which *n* = 180). An additional 10% (rounded to *n* = 20) was added to account for potential later exclusions (e.g., missing data, poor image quality). Population groups were categorised into broad ethnic groups as follows: (i) East Asian (Japanese, Korean, and Chinese), (ii) mestizo (Mexican and Chilean) and Hawaiian, (iii) South Asian (Indian and Malay), (iv) white (European and of European descent), (v) Eastern Mediterranean (Iranian, Turkish, Egyptian, Israeli Arab, and Israeli Jewish) and (vi) black (Kenyan, black South African, and black women in the UK and US). Mammograms were originally taken as part of organised screening (*n* = 13 studies), opportunistic or community-based screening (*n* = 8), mammography trials (*n* = 3), or research (*n* = 3). We also extracted the best estimate of the age-standardised incidence rate (ASR per 100,000) for breast cancer from Cancer Incidence in Five Continents, GLOBOCAN, or used another estimate [17,18] for each population group. From these ASR estimates, population groups were categorised as originating from a source population with low (ASR < 50), medium (ASR 50–69), or high (ASR ≥ 70) breast cancer incidence rate.

ICMD harmonised data from each study on sociodemographic and lifestyle factors, including height and weight to calculate body mass index (BMI, in kg/m^2^), ideally obtained at the time of mammography. For menopausal status, study-specific definitions were used, as provided in S1 Table. In brief, postmenopausal at the time of mammography was defined as no longer having menstrual periods (*n* = 7 studies) or no periods for the past 6 or 12 months (*n* = 15), self-reported postmenopausal (*n* = 3), or a combination of age, menstruation, hormone replacement therapy (HRT), and hysterectomy/oophorectomy history (*n* = 2). Perimenopausal women were excluded from ICMD sample selection in 10 studies, were not defined separately in 13 studies, and were included and categorised separately in 4 studies (2.7% of all women). For consistency, the latter were reclassified as premenopausal for analyses. Menopausal variables were ascertained at or after mammography for 22 studies. For 4 of the remaining studies, menopausal status was assumed not to have changed as it was ascertained less than 1 year before mammography, on average. For 1 study, in which risk factors were ascertained via questionnaire an average of 2.8 years before mammography, a premenopausal woman at the time of the questionnaire was considered postmenopausal at the time of mammography if her age at mammography exceeded the study’s mean age at menopause.

In ICMD, MD was read centrally in Cumulus [19] on digitised film-screen, raw digital, or processed digital images, by 1 of 3 experienced readers (VM, IdSS, NB). For each woman, 1 image (craniocaudal or mediolateral oblique view) was read. Images were randomly allocated to batches, and batches were randomly allocated to readers, as previously described [16]. Readings included 3% within-reader, within-batch repeats and 5% between-reader repeats. MD measures obtained were dense area, non-dense area, and breast area (dense area + non-dense area), all in square centimetres, and PD (PD = 100 × dense area/breast area). PD and dense and non-dense areas that were read on processed images were first corrected to a raw-image equivalent using published equations [20].

After excluding poor-quality images and suspected tumours (*n* = 288), women with no BMI data (*n* = 2) or with extreme BMIs (>3.8 SD from her population group mean, *n* = 42), 11,423 women were included, 11,232 of whom had PD data, 11,375 dense-area readings, and 11,184 non-dense-area and breast-area readings. Differences in the numbers of women with different MD metrics arose because, in some mammograms, dense area could be estimated but the breast edge was not visible (thus PD, non-dense area, and breast area were missing), and, for 78 images from 1 study, PD could be estimated, but uncertainties in the original pixel size prevented the calculation of absolute areas.

### Statistical methods

We analysed PD and dense, non-dense, and breast area after square-root transforming each measure to improve normality of residuals. The use of the square-root transformation of MD in regression models means that absolute differences in areas are smaller at the lower end of the PD range. For example, an increase in √PD from 4 to 5 (change in √PD of 1) is, on the original scale, an increase from 4^2^ to 5^2^, or an absolute increase of 9 (from 16 to 25), but the same change in √PD of 1 from 7 to 8 is, on the original scale, an increase from 7^2^ to 8^2^, or an absolute increase of 15 (from 49 to 64). To aid the interpretation on the square-root scale, √MD can be interpreted by considering areas as squares, thus regression model beta-coefficients represent differences in the width, in centimetres, of the side of the square. Similarly, as PD (percent) is the dense area per 100 cm^2^, √PD is the width of a dense square within a 10 cm × 10 cm square, and therefore beta-coefficients for √PD represent differences in the dense square width, within a 10 cm × 10 cm square.

We examined cross-sectional differences in √MD measures by age and menopausal status using 2 approaches. First, we examined associations by population group and combined estimates using a random effects meta-analysis. Population group estimates were obtained from a multi-level linear regression model to account for repeat MD readings between readers and between batches for the same woman. The percentage of variation in regression estimates across population groups that was due to heterogeneity was examined using the *I*^2^ statistic [21], for which percentages of 0%, <30%, 30%–60%, and >60% were roughly considered as no, low, moderate, and large heterogeneity, respectively. Second, we pooled all ICMD data and analysed them in a single model by extending the multi-level model to a third level to account for within-population-group correlations. For both approaches, explanatory variables included fixed effects for the 2 primary variables of interest, age (linear) and menopausal status (binary), as well as BMI (linear + quadratic), ever use of HRT (coded as never, past, current, ever [some studies did not distinguish past and current use], or unknown), parity (continuous), age at first birth (categorical: <20, 20 to <25, 25 to <30, ≥30 years), and reader (categorical). All analyses were based on participants with non-missing data for all variables, as completeness was high (>99%), as per eligibility for inclusion. For pooled analyses (the second approach), factors with between-study variation were additionally adjusted for, i.e., mammogram view and image type. The pooled analysis was also used to examine associations with age in 5-year bands, and linear associations with age were compared to the best-fitting fractional polynomial models.

Interactions of age and menopausal status with demographic and lifestyle factors were tested using likelihood ratio tests. We also examined effect modification by ‘breast area for BMI’, a proxy for a woman’s breast size independent of her BMI, which we generated as the residual of the regression of √breast area on BMI (linear and quadratic terms) and mammography view. Sensitivity analyses excluding women with very early or late menopause, those under 40 or over 70 years, and women with self-reported versus measured BMI were also conducted.

## Results

**Fig 1:**
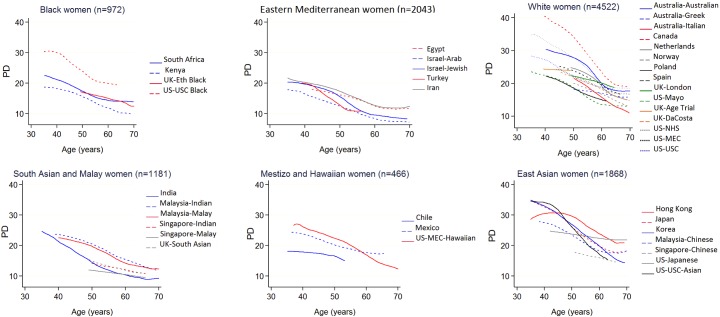
Polynomial smoothed curves of the crude association of percent mammographic density with age, for each population group within broad ethnic groups. The broad ethnic groups are organised from largest (black women) to smallest (East Asian women) average breast area for BMI. Full names and details of studies/population groups presented in this figure are provided in S1 Text). Adjustments: none. PD, percent mammographic density.

The 11,423 ICMD women ranged in age from 35 to 85 years at mammography, with at least 500 women in each 5-year age category from 35 to 70 (Table 1). Mean age at mammography was 45.6 (SD 4.9) in premenopausal women and 57.5 years (SD 6.4) in the 59% of women who were postmenopausal. East Asian women had both the smallest mean BMI and the smallest mean breast area for BMI (√breast area 0.2 to 1.2 cm smaller than ICMD average) (S2 Fig), and black women had the largest mean breast area for BMI (√breast area 0.5 to 1.3 cm larger than ICMD average). Crude trends for PD and dense area with age were inverse, held across most population groups, and appeared steeper around age 50 years than at either extreme of the age range studied (Figs 1 and S3).

**Table 1:** Characteristics of ICMD participants by age: Menopausal status, BMI, and measures of mammographic density.

Characteristic	Age at mammography (years)								
	35–39	40–44	45–49	50–54	55–59	60–64	65–69	≥70	All
Number of women, by broad ethnic group (ordered by decreasing breast area for BMI)*									
All	502	1,639	2,045	2,884	1,984	1,451	722	196	11,423
Black	67	139	159	251	176	136	34	29	991
Eastern Mediterranean	89	423	408	516	244	193	140	49	2,062
White	74	476	896	1288	844	647	345	69	4,639
South Asian and Malay	17	135	155	286	310	219	52	7	1,181
Mestizo and Hawaiian	121	134	102	122	51	30	23	1	584
East Asian	119	324	304	400	347	216	129	32	1,871
**Woman characteristics: mean (SD) unless stated otherwise**									
Age at mammography (years)	37.7 (1.4)	42.1 (1.5)	47.3 (1.5)	52.1 (1.5)	57.1 (1.5)	62.1 (1.4)	66.8 (1.4)	73.2 (3.6)	52.6 (8.2)
Percent postmenopausal^†^	3.2	5.9	18.5	66.9	96.5	99.7	99.6	99.0	58.6
Age at menopause (years)	39.8 (9.9)	41.7 (7.5)	44.2 (5.4)	47.3 (4.9)	49.0 (5.1)	49.1 (5.3)	49.1 (5.4)	47.8 (5.2)	48.1 (5.5)
Parity	2.2 (1.3)	2.3 (1.4)	2.3 (1.6)	2.5 (1.7)	2.8 (1.8)	3.2 (2.1)	3.4 (2.4)	3.4 (2.6)	2.6 (1.8)
Age at first birth, parous women	24.3 (4.9)	25.2 (5.4)	24.9 (5.3)	24.1 (5.1)	24.0 (5.1)	23.6 (4.8)	23.8 (4.9)	24.1 (5.6)	24.3 (5.2)
Percent HRT ever use	5.4	9.3	13.1	25.4	32.6	34.7	44.0	39.8	24.3
BMI^‡^ (kg/m^2^)	25.5 (5.4)	26.2 (5.3)	26.8 (5.8)	27.3 (5.5)	27.2 (5.2)	27.2 (5.4)	27.5 (5.6)	26.7 (5.8)	26.9 (5.5)
**Mammographic measures: mean (SD)**									
Percent density (%)	29.9 (15.4)	27.9 (15.0)	27.4 (15.0)	22.5 (13.8)	18.7 (12.3)	16.9 (11.8)	16.3 (12.3)	17.9 (14.9)	22.6 (14.4)
Dense area (cm^2^)	37.7 (20.9)	37.2 (21.3)	36.2 (21.6)	31.8 (21.2)	27.0 (18.6)	25.0 (17.6)	24.2 (18.7)	26.5 (18.4)	31.3 (20.8)
Breast area (cm^2^)	141.9 (68.8)	149.6 (71.3)	148.9 (72.1)	156.9 (70.7)	159.2 (65.3)	164.7 (71)	168.2 (72.7)	177.5 (77.3)	156.3 (70.7)
Non-dense area (cm^2^)	104.3 (65.1)	112.5 (67.6)	112.6 (68.0)	125.2 (67.5)	132.3 (64.2)	139.8 (69.1)	144.0 (70.7)	151.0 (76.8)	125.0 (68.5)
Breast area for BMI^‡^ (cm)	0.13 (1.82)	0.12 (1.96)	−0.10 (1.88)	−0.05 (1.94)	−0.01 (1.86)	0.06 (2.01)	0.00 (1.98)	0.51 (2.02)	0.00 (1.93)

*Ethnic groups include women of this ethnicity from the following countries. Black: Kenya, South Africa, US, UK. Eastern Mediterranean: Egypt, Iran, Israel (Arab), Israel (Jewish), Turkey. White: Australia (Australian, Greek, and Italian), Canada, Netherlands, Norway, Poland, Spain, UK, US. South Asian and Malay: India, Malaysia (Indian), Malaysia (Malay), Singapore (Indian), Singapore (Malay), UK. Mestizo and Hawaiian: Chile, Mexico, Hawaii. East Asian: Hong Kong, Japan, US (Japanese), Korea, Malaysia (Chinese), Singapore (Chinese).

^†^At the time of mammography.

^‡^Difference in square-root breast area adjusted for BMI compared to ICMD average.

BMI, body mass index; HRT, hormone replacement therapy; ICMD, International Consortium on Mammographic Density; SD, standard deviation.

### Menopause

**Fig 2:**
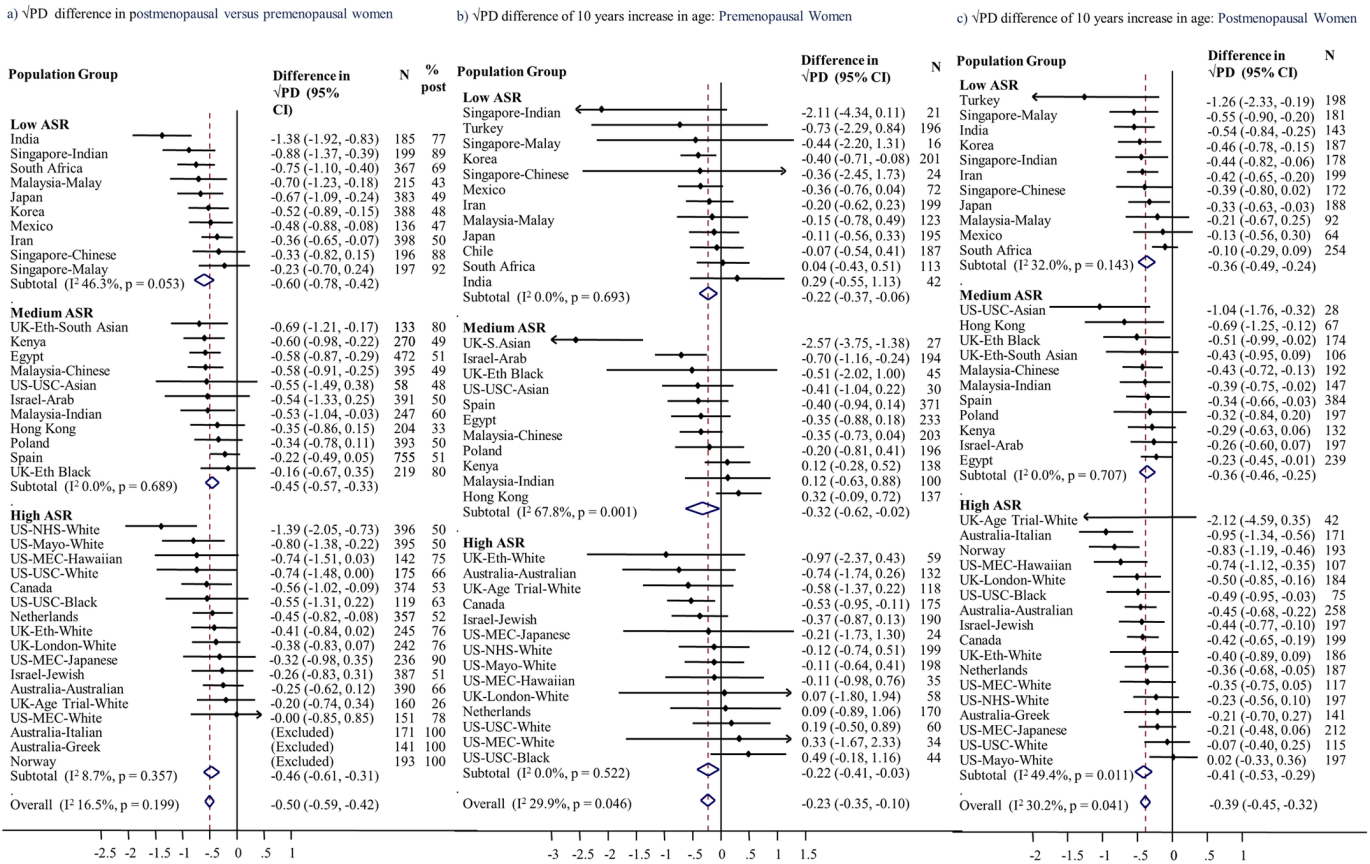
Association of square-root percent mammographic density with menopausal status and age. Associations of square-root percent density, by population group, with (a) menopausal status, (b) age among premenopausal women, and (c) age among postmenopausal women, meta-analysed overall and by ASR in source population (low, medium, high). Associations are adjusted for age (for [a] only), BMI, BMI2, parity, age at first birth, HRT use (never, current, former, ever, unknown), mammography view, and MD reader. Full names and details of studies/population groups presented in this figure are provided in S1 Text. Chile is excluded from (a) and (c) as all women were premenopausal. Norway, Australia (Greek), and Australia (Italian) were not included in (a) and (b) as all women were postmenopausal. Turkey was excluded from (a) as selection of women implied that age completely determined menopausal status. ASR, age-standardised incidence rate; BMI, body mass index; CI, confidence interval; HRT, hormone replacement therapy; MD, mammographic density; PD, percent mammographic density.

Menopausal differences showed no or low inconsistency across population groups for PD (*I*^2^ = 16.5%) and dense (*I*^2^ = 26.5%), non-dense (*I*^2^ = 0%), and breast area (*I*^2^ = 0%) (Figs 2A, S4A, S5A and S6A). The combined effect estimates for the menopausal difference showed lower PD and smaller dense area in postmenopausal compared to premenopausal women of the same age, but larger non-dense area and slightly larger breast area. These differences did not differ by ASR category (Fig 2A; *p* = 0.31 for PD). The findings from the pooled analyses closely matched the meta-analytic results, but confidence intervals (CIs) were narrower (Tables 2 and S2): the √PD difference was −0.46 cm (95% CI: −0.53, −0.39), which was driven by a smaller √dense area (difference −0.55 cm [95% CI: −0.65, −0.45]) and larger √non-dense area (difference +0.32 cm [95% CI: 0.21, 0.43]). However, in contrast to the meta-analysis combined estimate, breast area was not significantly larger in postmenopausal compared to premenopausal women (0.04 cm [95% CI: −0.07, 0.14]) (S2 Table).

**Table 2:** Difference in square-root mammographic density measures in postmenopausal compared to premenopausal women and by time since menopause: Overall and in subgroups (pooled analyses).

Variable	Number of women		Post- versus premenopausal difference*			
			Percent density		Dense area	
	Premenopausal	Postmenopausal	Difference (dense width [cm] in 10 × 10 cm square)	95% CI or *p*-value	Difference (dense square width [cm])	95% CI or *p*-value
**All women**			−0.46	−0.53, −0.39	−0.55	−0.65, −0.45
**Parity**						
Nulliparous	507	612	−0.30	−0.46, −0.14	−0.43	−0.64, −0.22
Parity 1–2	2,292	2,564	−0.46	−0.55, −0.37	−0.49	−0.61, −0.37
Parity 3–4	1,439	2,349	−0.50	−0.61, −0.40	−0.63	−0.76, −0.49
Parity ≥ 5	296	956	−0.53	−0.71, −0.35	−0.75	−0.99, −0.51
*p*-interaction				0.11		0.06
**Age at first birth (years)**^†^						
<20	612	1,231	−0.63	−0.77, −0.49	−0.84	−1.02, −0.65
20to <25	1,307	2,313	−0.47	−0.57, −0.36	−0.57	−0.71, −0.43
25to <30	1,382	1,658	−0.51	−0.62, −0.40	−0.55	−0.70, −0.41
≥30	730	669	−0.36	−0.51, −0.22	−0.30	−0.50, −0.11
*p*-interaction				0.04		<0.001
**HRT**^‡^						
Never	3,223	3,654	−0.49	−0.57, −0.41	−0.56	−0.67, −0.45
Past	137	673	−0.50	−0.74, −0.26	−0.69	−0.88, −0.50
Current	120	825	−0.11	−0.37, 0.14	−0.18	−0.51, 0.16
Ever	26	436	−0.29	−0.81, 0.23	−0.18	−0.86, 0.50
Missing	1,028	893	−0.46	−0.60, −0.32	−0.69	−0.88, −0.50
*p*-interaction				0.08		0.07
**BMI (kg/m**^**2**^**)**						
<20	305	362	−0.58	−0.79, −0.38	−0.35	−0.62, −0.07
20 to <25	1,816	2,223	−0.54	−0.64, −0.44	−0.47	−0.60, −0.34
25 to <30	1,355	2,200	−0.41	−0.52, −0.31	−0.54	−0.67, −0.40
30 to <35	702	1,114	−0.48	−0.61, −0.35	−0.76	−0.93, −0.59
≥35	356	582	−0.21	−0.38, −0.03	−0.62	−0.85, −0.39
*p*-interaction				0.005		0.02
**Breast area for BMI quintiles**						
1	898	1,288	−0.37	−0.49, −0.25	−0.26	−0.41, −0.10
2	939	1,281	−0.42	−0.54, −0.30	−0.39	−0.55, −0.24
3	935	1,256	−0.45	−0.57, −0.33	−0.53	−0.69, −0.38
4	872	1,311	−0.46	−0.58, −0.34	−0.67	−0.83, −0.52
5	871	1,316	−0.59	−0.72, −0.47	−1.00	−1.16, −0.85
*p*-interaction				0.06		<0.001
**Ethnic group**						
Black	342	628	−0.30	−0.48, −0.12	−0.60	−0.88, −0.32
Eastern Mediterranean	937	944	−0.46	−0.59, −0.32	−0.63	−0.84, −0.42
White	1,774	2,777	−0.43	−0.53, −0.33	−0.43	−0.56, −0.30
South Asian and Malay	327	834	−0.75	−0.93, −0.57	−0.72	−0.99, −0.44
Mestizo and Hawaiian	297	179	−0.46	−0.77, −0.15	−0.39	−0.77, −0.01
East Asian	810	1,041	−0.48	−0.62, −0.34	−0.39	−0.58, −0.20
*p*-interaction				0.01		0.15
Years since menopause**						
0–1.9	—	548	0		0	
2–4.9	—	1,133	−0.05	−0.18, 0.08	−0.07	−0.24, 0.11
5–9.9	—	1,664	−0.24	−0.37, −0.11	−0.27	−0.44, −0.10
10–14.9	—	1,295	−0.30	−0.44, −0.16	−0.36	−0.54, −0.17
≥15	—	1,375	−0.29	−0.45, −0.14	−0.37	−0.57, −0.17
*p*-difference				<0.001		<0.001
**Trend in years since menopause, per 10 years**		6,015	−0.10	−0.17, −0.04	−0.13	−0.22, −0.05

Estimated from a multi-level model on pooled data, with level 3 = population group, level 2 = woman, and level 1 = MD reading. All models are adjusted for age, BMI, BMI2, mammogram view, image type, reader, parity, age at first birth, and HRT use.

*Difference in square-root MD (postmenopausal minus premenopausal) in each subgroup was generated from menopause-by-subgroup interaction term.

^†^Parous women only.

^‡^HRT categories reflect the different levels of HRT use details available for different studies; women do not appear in more than 1 category.

**Postmenopausal women only, age-adjusted. N = number of women with percent density measurement (N for other measures is −0.4% to +1.3% different).

BMI, body mass index; CI, confidence interval; HRT, hormone replacement therapy; MD, mammographic density.

The menopause-associated PD difference held across the subgroups of women examined. Where effect modification was present, the associations differed in magnitude, but not direction (Tables 2 and S2). For PD and dense area, interactions were found with parity, age at first birth, HRT use, BMI, and breast area for BMI (e.g., tests for interaction with PD, *p* = 0.11, 0.04, 0.08, 0.005, and 0.06, respectively; Table 2), and were independent of each other. The magnitude of the menopause-associated difference in dense area, and consequently in PD, was larger in women with lower breast cancer risk profiles, i.e., larger in women who had had an early age at first birth (difference in √dense area of −0.84 cm if <20 years and −0.30 cm if ≥30 years at first birth; Table 2), in women with higher parity, and in never and past HRT users (and null in current users). Across the broad ethnic groups, menopause–MD associations were of a similar magnitude, except for a larger decline in √PD (−0.75 cm [95% CI: −0.93, −0.57]) in the South Asian and Malay group. For effect modification by BMI, in contrast to the aforementioned interactions, whose effects on PD and dense area were in similar directions, the largest menopause-associated differences in dense area occurred in women with a BMI ≥ 30 kg/m^2^, whilst these women tended to have the smallest menopause-associated differences in PD (Table 2). Amongst women with a BMI ≥ 35 kg/m^2^, postmenopausal women had smaller breast areas than premenopausal women, in contrast to findings of no difference in other categories (S2 Table).

### Age

Meta-analyses (Fig 2B and 2C) and pooled analyses (Table 3) showed very similar age–MD associations. For a 10-year increase in age, the difference in √PD was −0.24 cm among premenopausal women and −0.38 cm among postmenopausal women (*p* for interaction by menopausal status = 0.15). These associations reflect similar inverse √dense area–age associations (−0.27 and −0.32 cm for pre- and postmenopausal women). However, whereas in premenopausal women PD differences occurred without a difference in breast area, in postmenopausal women √breast area was 0.34 cm (95% CI: 0.24, 0.39) larger per 10-year age increase (S3 and S4 Tables; S5B and S5C Fig). PD–age associations had low inconsistency across population groups (*I*^2^ ~ 30%) and across ASR categories; in postmenopausal women, all point estimates were negative.

**Table 3:** Difference in square-root mammographic density measures with a 10-year difference in age, in pre- and postmenopausal women.

Variable	Premenopausal women					Postmenopausal women				
	*N*	Percent density (width [cm] of dense square in a 10 × 10 cm square area)		Dense area (width [cm] of dense square)		*N*	Percent density (width [cm] of dense square in a 10 × 10 cm square area)		Dense area (width [cm] of dense square)	
		Difference	95% CI or *p*-value	Difference	95% CI or *p*-value		Difference	95% CI or *p*-value	Difference	95% CI or *p*-value
**Age, per 10 years**	4,534	−0.24	−0.34, −0.14	−0.27	−0.41, −0.14	6,481	−0.38	−0.44,−0.33	−0.32	−0.39, −0.24
*p*-interaction by menopausal status								0.15		0.67
**Age category (years)**	4,456					6,466				
35 to <40	464	0.09	−0.05, 0.22	0.10	−0.09, 0.28	—	—	—	—	—
40 to <45	1,478	0	—	0	—	96	0.38	0.10, 0.66	0.33	−0.04, 0.70
45 to <50	1,591	−0.07	−0.17, 0.02	−0.15	−0.28, −0.02	365	0.13	−0.02, 0.28	0.00	−0.20, 0.19
50 to <55	923	−0.23	−0.36, −0.10	−0.28	−0.46, −0.11	1,857	0	—	0	—
55 to <60	—	—	—	—	—	1,867	−0.24	−0.33, −0.16	−0.21	−0.32, −0.11
60 to <65	—	—	—	—	—	1,407	−0.46	−0.55, −0.37	−0.41	−0.53, −0.29
65 to <70	—	—	—	—	—	692	−0.58	−0.70, −0.46	−0.53	−0.68, −0.37
≥70	—	—	—	—	—	182	−0.67	−0.88, −0.46	−0.57	−0.85, −0.29
*p*-difference			<0.001		0.002			<0.001		<0.001
Difference per 10-year increase in age by broad ethnic group*										
Black	342	−0.08	−0.37, 0.22	−0.23	−0.63, 0.16	628	−0.17	−0.31, −0.02	−0.19	−0.33, −0.06
Eastern Mediterranean	937	−0.20	−0.45, 0.04	−0.31	−0.64, 0.02	944	−0.32	−0.47, −0.18	−0.42	−0.53, −0.31
White	1,774	−0.36	−0.53, −0.18	−0.36	−0.59, −0.13	2,777	−0.42	−0.51, −0.34	−0.32	−0.40, −0.24
South Asian and Malay	327	−0.27	−0.61, 0.06	−0.31	−0.75, 0.12	834	−0.49	−0.65, −0.32	−0.49	−0.64, −0.33
Mestizo and Hawaiian	297	−0.01	−0.34, 0.32	−0.16	−0.58, 0.25	179	−0.55	−0.86, −0.25	−0.25	−0.47, −0.03
East Asian	810	−0.22	−0.41, −0.02	−0.16	−0.42, 0.11	1,041	−0.38	−0.52, −0.25	−0.22	−0.33, −0.11
*p*-interaction			0.43		0.90			0.02		0.34

Estimated from a multi-level model of pooled data, with level 3 = population-group, level 2 = woman, and level 1 = MD reading. All models are adjusted for BMI, BMI2, mammogram view, image type, reader, parity, age at first birth, and HRT use.

*Age-associated differences in each subgroup were generated from age-by-subgroup interaction terms. N = number of women with percent density measurement (N for other measures differs by −0.5% to +1.8%).

BMI, body mass index; CI, confidence interval; HRT, hormone replacement therapy; MD, mammographic density.

**Fig 3:**
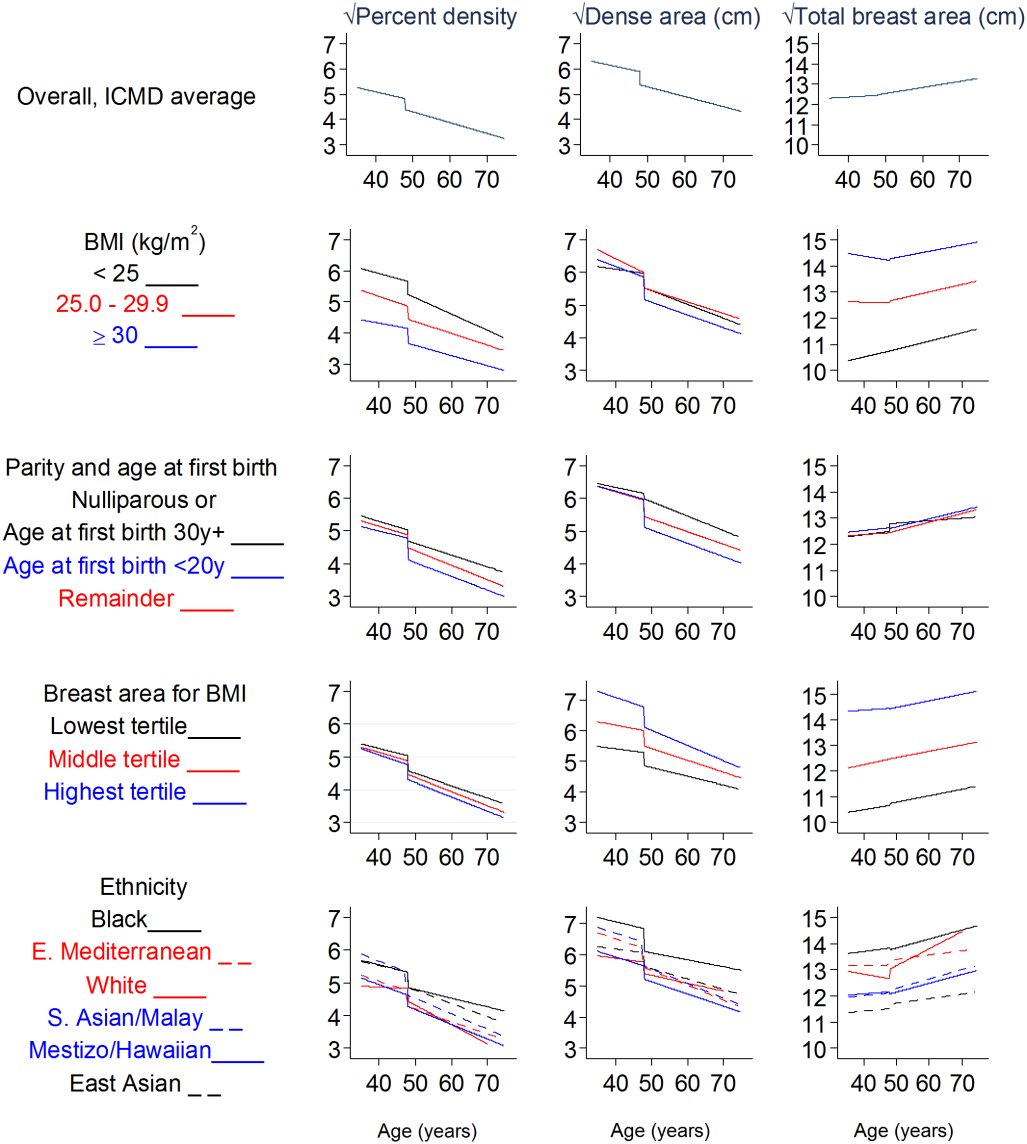
Modelled associations of square-root percent density, dense area, and total breast area with age and menopausal status, overall and by subgroups. Square-root dense/breast area is the width of a square representing the dense/breast area; square-root PD is the width of a dense-area square within a 10 cm × 10 cm square. All models are adjusted for age, BMI, BMI2, parity, age at first birth, HRT use (never, ever, past, current, not known), MD reader, image type, and mammography view. BMI, body mass index; CI, confidence interval; ICMD, International Consortium on Mammographic Density;HRT, hormone replacement therapy; MD, mammographic density.

Fig 3 shows overall and group-specific fitted associations of MD measures with age, including a main effect of menopausal status and a menopause-by-age interaction term to allow for different age associations (slopes) in pre- and postmenopausal women. Predictions were made by age, assuming premenopausal up to age 50 years and postmenopausal thereafter; thus, the change at age 50 years represents menopausal difference. In contrast to the multiple effect modifiers found for the PD–menopause difference, MD–age associations did not differ substantially between women grouped by most reproductive factors (S3 and S4 Tables; Fig 3). In Fig 3 the few exceptions include a smaller age-associated PD difference in current and ever users of HRT compared to never users (*p* for interaction = 0.045; S4 Table), which appeared to be driven by a differential association with dense area (*p* = 0.007) and not with non-dense or breast area (*p* = 0.46 and 0.23, respectively). Additionally, among premenopausal women, there was some evidence that the inverse √dense area–age association was stronger in nulliparous than parous women (−0.68 versus −0.25 overall, *p* for interaction = 0.06), and whilst there were no differences by ethnicity in premenopausal women, in postmenopausal women the PD–age slope was shallowest for black women (Fig 3).

Further examination of MD–age associations using the best-fitting flexible fractional polynomials revealed that such non-linear models were not a better fit than the simplest linear models for PD (*p* = 0.42), dense area (*p* = 0.90), and breast area (*p* = 0.09) in premenopausal women and for all 4 MD measures in postmenopausal women (*p* = 0.23, 0.66, 0.53, and 0.91 for PD, dense, non-dense, and total breast area, respectively), thus suggesting that there is no plateauing of MD within this age range. For non-dense area in premenopausal women, a fractional polynomial model had a better fit than a linear model (*p* = 0.008), as the positive association with age was not seen until around age 45 years. In sensitivity analyses, the PD–age association in premenopausal women was attenuated from −0.24 to −0.18 if older premenopausal women (>50 years) were excluded, and postmenopausal PD–age associations were stronger (increased from −0.38 to −0.47) when women over age 65 years were excluded (estimates are provided here and do not appear in tables).

### Ageing and the menopause

In postmenopausal women, there is sufficient variability in age at menopause (IQR 45 to 52 years) to analyse the impact of age-adjusted years since menopause, which was independently associated with √PD (−0.10 per 10 years [95% CI: −0.17, −0.04]), an effect that was present up to 15 years after menopause (Table 2). After adjusting for years since menopause, the PD–age association reduced from −0.38 to −0.29 per 10 years, but remained significant.

## Discussion

### Main findings

Across diverse populations of breast-cancer-free women worldwide, we assessed how MD is related to age and menopausal status. Overall, despite inevitable differences between contributing studies, we found consistency in the direction of associations of age and menopausal status with PD and its component tissues across ICMD women. Assuming that inferences from these cross-sectional data reflect within-woman changes, the greatest reduction in MD occurred upon menopausal transition, when dense area declined from a mean of 16.0 to 12.5 cm^2^, equivalent to 15 to 20 years of ageing. This difference in PD between post- and premenopausal women of the same age was present up to 15 years after the menopause and may be greater in women with lower breast cancer risk profiles (early age at first birth, high parity). PD also decreased with increasing age both among premenopausal and postmenopausal women. In premenopausal women, these declines were entirely due to breast compositional changes, i.e., due to a decline in dense area and an equal but opposite increase in non-dense area. However, in postmenopausal women, PD reductions were also accompanied by increasing breast area and continued beyond 70 years of age.

### Comparison to other studies

The large decrease in MD at menopause after adjusting for age is largely consistent with previous observations in westernised countries, either from within-woman or between-woman comparisons [2,8–10,12,15]. Boyd et al. [2] examined differences in breast area before and after menopause and found no significant change, indicating that lower PD after menopause may be due a compositional change alone. Only 1 longitudinal study reported a decline in PD at menopause that was non-significant, although in this study the largest age-related decline did occur around the age of menopause (between ages 45 and 55 years) [11].

Comparisons of age-related changes have been more variable. In ICMD, we found no strong evidence of different PD–age or dense area–age slopes in pre- and postmenopausal women, in agreement with the full longitudinal data from 1 of the ICMD studies, the Melbourne Collaborative Cohort Study/Australian Breast Cancer Family Registry [22], but differing from studies that reported steeper MD declines in either premenopausal [14] or postmenopausal women [9,11]. In addition, there was no strong evidence of non-linear MD–age trends in ICMD, but 3 longitudinal studies have found that PD (and dense area, where examined) declines most rapidly around ages 45 to 55 or 60 years [8,11,12], including the Melbourne study, which also observed a possible plateau after 65 years of age [11,12,22].

### Biological plausibility

The remarkable consistency of the age and menopausal effects on MD across diverse female populations spanning all continents suggests that these associations reflect a biological process common to all women. The extent of the decline in MD may be modified by ‘external’ factors, such as reproductive or other lifestyle or environmental factors. These observations lend credibility to the hypothesis that the sharp decline in MD over the menopause is a phenotypic marker of the decline in the rate of breast tissue ageing [4], which results in Clemmesen’s hook in the age–breast cancer incidence curves across the world. However, this simple parallel ignores the well-established molecular and aetiological heterogeneity of breast cancer [5]. Notably, although it has previously been hypothesized that the hormonal drivers of PD would result in it being a stronger risk marker for oestrogen receptor (ER)–positive than ER-negative breast cancer, a large differential in the MD-breast cancer association by tumour subtype does not appear to be present [23]. This finding should perhaps not be surprising, if the menopausal decline in PD, observed universally here, is related to the incidence curves of breast cancer with age, which feature a much stronger menopausal decline in the age-acceleration of incidence rates for ER-negative tumours than for ER-positive tumours [5,24]. At the tissue level, changes involving the dense area arise from changes in stromal tissue and/or in epithelial tissue, whilst changes in PD are additionally affected by changes in adipose tissue in the breast. Declining dense area likely reflects involution of terminal duct lobular units. X-rays of histological tissue show that these units have raised concentrations of radiologically dense areas and that these areas also decline with age [25]. Two additional observations link MD, terminal duct lobular units, and breast cancer risk: breast carcinoma arises within epithelial tissue and the radiologically dense tissue seen on a mammogram [26], and, within cohorts with benign breast disease, greater terminal duct lobular unit involution is predictive of subsequent breast cancer risk [27].

At a systemic level, a hormonal pathway is most certainly implicated in MD changes that occur during the menopausal transition. Several reproductive hormones decline at different stages of the transition [28], some of which may contribute to the MD decline. Combined exogenous oestrogen and progesterone are known to increase PD [29], whilst in premenopausal women, endogenous oestrogen levels are positively associated with PD [30]. The shallower age-related declines in PD in premenopausal women compared to postmenopausal women in our study may reflect similar hormonal pathways. Further, in postmenopausal women only, an increase in breast area also contributed to the decreasing PD. This is likely to reflect a true increase in breast volume as breast area and thickness were positively correlated in ICMD (*r* = 0.18).

### Strengths and limitations

The present study is greatly enhanced by the heterogeneity of the study populations included in ICMD, in terms of a wide range of BMI values, reproductive characteristics, ages (40-year age range), and ethnicities and their associated range of breast sizes and composition, making this, to our knowledge, the largest multi-ethnic, multi-country study of MD to date. The consistent findings are likely to reflect intrinsic biological processes and should therefore apply to the general population of women worldwide. Our separate analyses of tissue components revealed important differences in the drivers of changes in breast tissue composition at different life stages. However, inherent to the consortial nature of ICMD is the fact that we relied on existing data; thus, some sample sizes were smaller, not all studies included women spanning the full age range, and there is variability in image types and exposure variable definitions. Menopause was included as binary variable due to the limitations of the data, but may occur over many months or years. The definition of menopausal status also varied, from simple self-reports to complex algorithms, measured at or some time before or after mammography. Potential misclassification bias might attenuate the estimate of the menopausal effect but is unlikely to affect within-population group estimates of the menopause difference. Additionally, including some perimenopausal women would also underestimate a menopausal effect. In the 31% of women with self-reported anthropometry, possible underreporting of weight and overestimation of height, particularly in older women, would lead to an underestimation of BMI [31]; thus, part of the increase of breast area postmenopausally may be due to incomplete adjustment for BMI. In addition, the validity of BMI as a measure of total body adiposity across such diverse populations is uncertain.

Women in ICMD were recruited from a range of settings, including some that were not population-based screening; thus, there is likely an overrepresentation of symptomatic or high-risk women. Nevertheless, the present findings rely on the representativeness of the associations and not of the sample populations; thus, any lack of representativeness of study populations would affect results only if there was an age-related pattern to representativeness. In a similar manner, cross-sectional associations may only partially reflect within-woman changes, as birth cohort effects on MD, through factors unadjusted for, may confound associations. These findings should be verified with longitudinal data in which within-woman changes can be examined.

### Implications

There are broader implications of the profile of MD with ageing. Similar to the reduced sensitivity of mammography in younger women than in older women in organised screening programs [32], because of the former group’s higher average breast density, this problem is also likely to affect the performance of mammography as part of the diagnostic work-up of symptomatic breast cancers. Low- and middle-income countries may be disproportionately affected by this issue, because of the young population structures and thus younger average age at breast cancer diagnosis. Further, if MD reflects the rate of breast tissue ageing, the parallel to Pike’s model suggests that cumulative MD to a given age is the pertinent marker of breast cancer risk. Indeed, cumulative density has been shown to be highly correlated with age-related breast cancer risk [11], and women whose MD decreases substantially over time may be at lower risk of breast cancer than those who maintain the same density [10,33]. More efficient and cost-effective stratified approaches to population-based breast cancer screening using MD-based risk scores have been suggested [34]. Placing a woman in a particular MD-based risk stratum would be aided by the fact that MD displays a high level of tracking within a woman over time, at least postmenopausally [12], but it is not known whether risk predictions might be improved if earlier life periods, before MD tracking is established, were included.

Finally, based on the current findings that MD is in general highest earlier in life, primary prevention efforts might best be targeted to younger ages, reducing breast density earlier in life, leading to a lower cumulative MD across life. Studies of breast density in girls or younger women that use MRI or dual-energy X-ray absorptiometry (DXA) imaging are fewer. They have found suggestions of increased breast density associated with greater birth size [35], duration of use of hormonal contraceptives [36], higher testosterone levels [37], and lower childhood BMI, independent of current BMI [38]. At adult ages, the potential actions needed to lower breast density for a reduction in breast cancer risk, apart from the known pregnancy-associated effects, include lowering alcohol intake [39], whilst the protective effect of physical activity does not appear to be mediated through MD [40]. Further, in high-risk women and in women with ductal carcinoma in situ, tamoxifen reduces breast cancer risk through a reduction in MD [41]. Given the relatively low uptake of such oral chemoprevention therapies, other risk-lowering options for women with high breast cancer risk and raised MD may come from the ongoing trials of oral low dose and topical tamoxifen [42]. The consistency of the MD–ageing associations internationally is suggestive of an intrinsic shared biology, which might also extend to other modifiable determinants of MD.

## Supporting information

S1 Fig Age profile of breast cancer incidence rates in a selection of 6 countries included in ICMD that span low to high incidence rates (y-axis on log scale). (DOCX)

S2 Fig Population-group-specific difference in square-root breast area compared to the ICMD average square-root breast area, adjusted for BMI (linear and quadratic) and mammography view and grouped by broad ethnic group. (DOCX)

S3 Fig Smoothed curves of square-root percent density, dense area, and breast area, for broad ethnic groups. Curves are crude associations (no adjustment for BMI or any other factors).
(DOCX)

S4 Fig Association of dense area with menopause and age. Associations of square-root dense area (cm), by population group, with (a) menopausal status, (b) age in premenopausal women, and (c) age in postmenopausal women, meta-combined overall and by low, medium, and high breast cancer incidence rate in the source population.
(DOCX)

S5 Fig Association of total breast area with menopause and age. Associations of square-root breast area (cm), by population group, with (a) menopausal status, (b) age in premenopausal women, and (c) age in postmenopausal women, meta-combined overall and by low, medium, and high breast cancer incidence rate in the source population.
(DOCX)

S6 Fig Association of non-dense area with menopause and age. Associations of square-root non-dense area (cm), by population group, with (a) menopausal status, (b) age in premenopausal women, and (c) age in postmenopausal women, meta-combined overall and by low, medium, and high breast cancer incidence rate in the source population.
(DOCX)

S1 Table Study-specific definitions of menopause. (DOCX)

S2 Table Difference in square-root mammographic density measures in postmenopausal women compared to premenopausal women, and by time since menopause: Overall and by subgroups. (DOCX)

S3 Table Difference in square-root mammographic density measures with a 10-year increase in age in premenopausal women: Overall and by subgroups. (DOCX)

S4 Table Difference in square-root mammographic density measures with a 10-year increase in age, in postmenopausal women: Overall and by subgroups. (DOCX)

S1 Text List of studies included, by broad ethnic group (decreasing order of breast area for BMI). (DOCX)

S1 STROBE Checklist (DOC)

## Abbreviations

ASR: age-standardised incidence rate
BMI: body mass index
CI: confidence interval
ER: oestrogen receptor
HRT: hormone replacement therapy
ICMD: International Consortium on Mammographic Density
MD: mammographic density
PD: percent mammographic density
SD: standard deviation
