# Supplementary material for: Mammographic density and ageing: A collaborative pooled analysis of cross-sectional data from 22 countries worldwide
Source: PLoS Med. 2017 Jun 30;14(6):e1002335. doi: 10.1371/journal.pmed.1002335 (PMC5493289; doi:10.1371/journal.pmed.1002335)
Supplement: S1 Fig — (DOCX) [file pmed.1002335.s001.docx]

**S1 Fig:** Age profile of breast cancer incidence rates in a selection of 6 countries included in ICMD that span low to high incidence rates (y-axis on log scale)


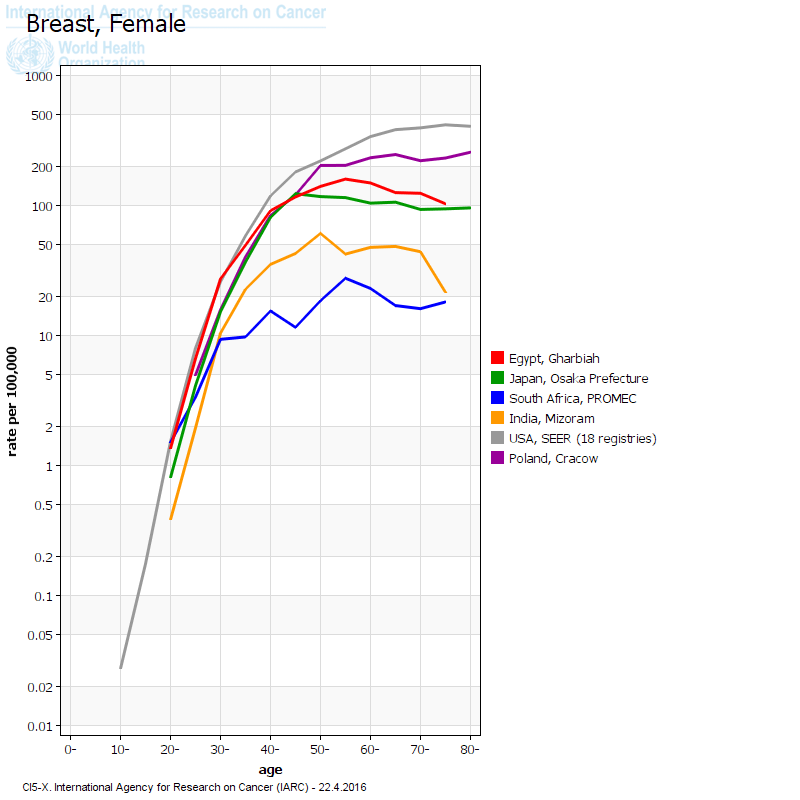


Source: Cancer Incidence in Five Continents, Volume X, International Agency for Research on Cancer
