# Supplementary material for: Mammographic density and ageing: A collaborative pooled analysis of cross-sectional data from 22 countries worldwide
Source: PLoS Med. 2017 Jun 30;14(6):e1002335. doi: 10.1371/journal.pmed.1002335 (PMC5493289; doi:10.1371/journal.pmed.1002335)
Supplement: S2 Fig — (DOCX) [file pmed.1002335.s002.docx]

**S2 Fig:** Population-group specific difference in square-root breast area compared to the ICMD-average square-root breast area, adjusted for BMI (linear and quadratic) and mammography view and grouped by broad ethnic group

**East Asian**

Japan

US-MEC-Japanese

Singapore-Chinese

Malaysia-Chinese

Hong Kong

Korea

US-USC-Asian

**South Asian & Malay**

Malaysia-Malay

India

Singapore-Malay

Singapore-Indian

Malaysia-Indian

UK-Eth-South Asian

**Mestizo & Hawaiian**

US-MEC-Hawaiian

Mexico

Chile

**White**

Netherlands

UK-Age Trial-White

Spain

UK-London-White

US-Mayo-White

US-NHS-White

UK-Eth-White

Australia-Australian

Australia-Italian

Poland

Australia-Greek

US-MEC-White

Canada

US-USC-White

Norway

**Eastern Mediterranean**

Iran

Turkey

Israel-Arab

Egypt

Israel-Jewish

**Black**

UK-Eth Black

Kenya

South Africa

US-USC-Black

**Population Group**

-1.21 (-1.34, -1.08)

-1.20 (-1.40, -1.01)

-0.90 (-1.08, -0.72)

-0.70 (-0.84, -0.57)

-0.50 (-0.68, -0.32)

-0.45 (-0.58, -0.32)

-0.22 (-0.83, 0.38)

-0.51 (-0.71, -0.30)

-0.47 (-0.68, -0.26)

-0.46 (-0.66, -0.26)

-0.21 (-0.42, 0.00)

0.26 (0.05, 0.46)

0.30 (-0.02, 0.61)

-0.42 (-0.75, -0.09)

-0.40 (-0.65, -0.15)

-0.07 (-0.31, 0.17)

-0.79 (-0.96, -0.62)

-0.53 (-0.82, -0.25)

-0.47 (-0.59, -0.35)

-0.44 (-0.64, -0.24)

-0.38 (-0.56, -0.20)

-0.03 (-0.21, 0.14)

0.10 (-0.12, 0.31)

0.11 (-0.05, 0.27)

0.21 (0.01, 0.41)

0.29 (0.10, 0.48)

0.32 (-0.03, 0.67)

0.34 (0.02, 0.66)

0.45 (0.27, 0.64)

0.72 (0.35, 1.08)

0.98 (0.61, 1.35)

0.36 (0.15, 0.57)

0.53 (0.36, 0.70)

0.54 (0.34, 0.74)

0.64 (0.45, 0.84)

0.87 (0.68, 1.06)

0.52 (0.26, 0.77)

0.63 (0.38, 0.87)

1.15 (0.87, 1.42)

1.26 (0.70, 1.82)

Difference (95% CI)

-1.21 (-1.34, -1.08)

-1.20 (-1.40, -1.01)

-0.90 (-1.08, -0.72)

-0.70 (-0.84, -0.57)

-0.50 (-0.68, -0.32)

-0.45 (-0.58, -0.32)

-0.22 (-0.83, 0.38)

-0.51 (-0.71, -0.30)

-0.47 (-0.68, -0.26)

-0.46 (-0.66, -0.26)

-0.21 (-0.42, 0.00)

0.26 (0.05, 0.46)

0.30 (-0.02, 0.61)

-0.42 (-0.75, -0.09)

-0.40 (-0.65, -0.15)

-0.07 (-0.31, 0.17)

-0.79 (-0.96, -0.62)

-0.53 (-0.82, -0.25)

-0.47 (-0.59, -0.35)

-0.44 (-0.64, -0.24)

-0.38 (-0.56, -0.20)

-0.03 (-0.21, 0.14)

0.10 (-0.12, 0.31)

0.11 (-0.05, 0.27)

0.21 (0.01, 0.41)

0.29 (0.10, 0.48)

0.32 (-0.03, 0.67)

0.34 (0.02, 0.66)

0.45 (0.27, 0.64)

0.72 (0.35, 1.08)

0.98 (0.61, 1.35)

0.36 (0.15, 0.57)

0.53 (0.36, 0.70)

0.54 (0.34, 0.74)

0.64 (0.45, 0.84)

0.87 (0.68, 1.06)

0.52 (0.26, 0.77)

0.63 (0.38, 0.87)

1.15 (0.87, 1.42)

1.26 (0.70, 1.82)

Difference (95% CI)

0

-2

-1

0

1

2

Difference in square-root breast area (cm)

Population-group mean difference in square-root breast area-for-BMI
