# Supplementary material for: Mammographic density and ageing: A collaborative pooled analysis of cross-sectional data from 22 countries worldwide
Source: PLoS Med. 2017 Jun 30;14(6):e1002335. doi: 10.1371/journal.pmed.1002335 (PMC5493289; doi:10.1371/journal.pmed.1002335)
Supplement: S3 Fig — Curves are crude associations (no adjustment for BMI or any other factors). (DOCX) [file pmed.1002335.s003.docx]

**S3 Fig:** Smoothed curves of square-root percent density (1^st^ row), dense area (2^nd^ row) and breast area (3^rd^ row), for broad ethnic groups. Curves are crude associations (no adjustement for BMI or any other factors)
