# Supplementary material for: Mammographic density and ageing: A collaborative pooled analysis of cross-sectional data from 22 countries worldwide
Source: PLoS Med. 2017 Jun 30;14(6):e1002335. doi: 10.1371/journal.pmed.1002335 (PMC5493289; doi:10.1371/journal.pmed.1002335)

**S6 Fig:** Associations of square-root non-dense area (cm), by population group, with (a) menopausal status, (b) age at pre-menopausal ages and (c) age at post-menopausal ages, meta-combined overall and by low, medium and high breast cancer incidence rate in the source population

All adjusted for BMI, BMI^2^, HRT use (never, current, former, ever), reader age at first birth, and parity. a) additionally adjusted for age.

Chile excluded from a) and c) as all women premenopausal. Norway, Australia-Greek and Australia-Italian excluded from a) and b) as all women postmenopausal. Turkey excluded from a) as age and menopausal status completely correlated.


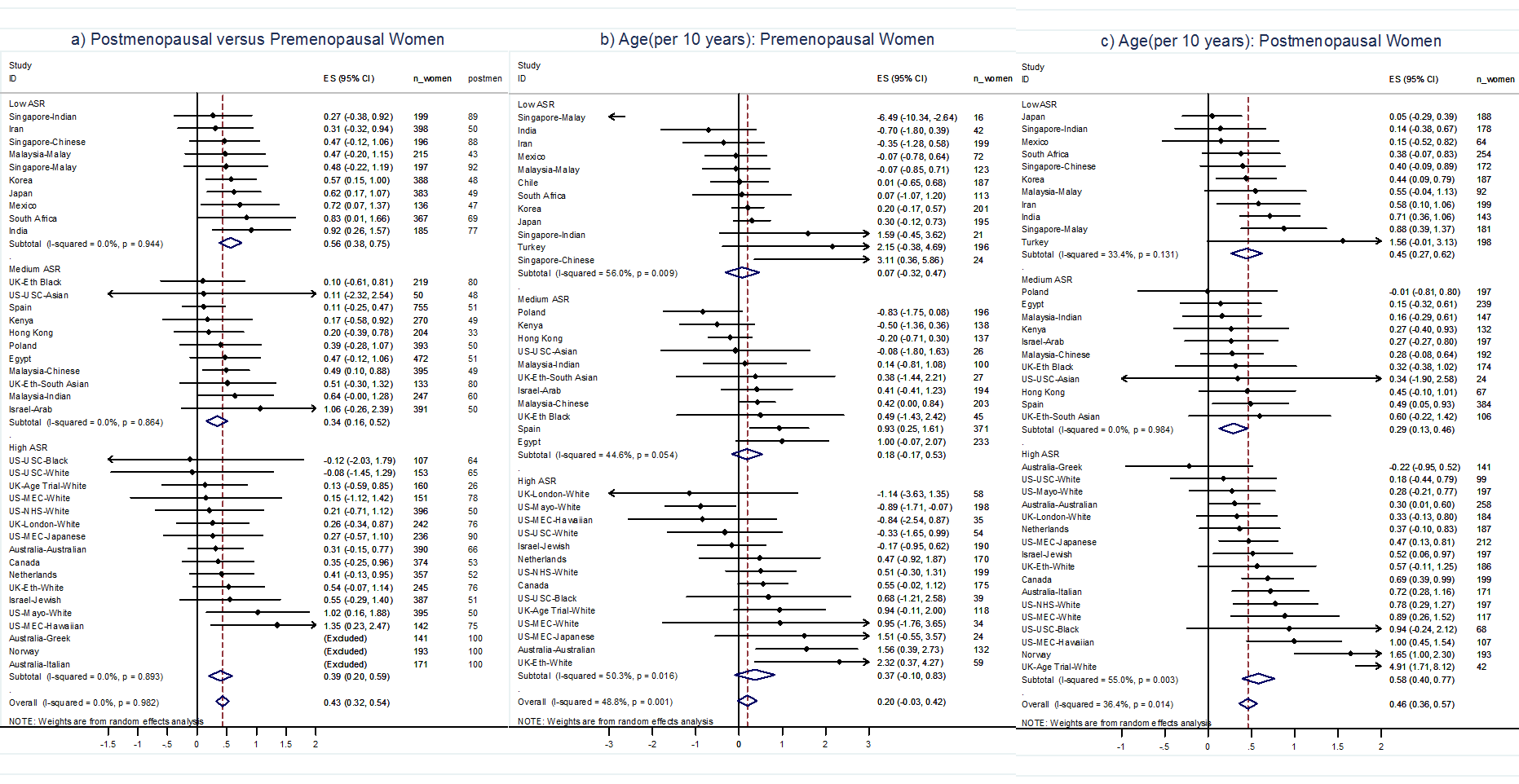

Supplement: S6 Fig — Associations of square-root non-dense area (cm), by population group, with (a) menopausal status, (b) age in premenopausal women, and (c) age in postmenopausal women, meta-combined overall and by low, medium, and high breast cancer incidence rate in the source population. (DOCX) [file pmed.1002335.s006.docx]
