# Supplementary material for: Mammographic density and ageing: A collaborative pooled analysis of cross-sectional data from 22 countries worldwide
Source: PLoS Med. 2017 Jun 30;14(6):e1002335. doi: 10.1371/journal.pmed.1002335 (PMC5493289; doi:10.1371/journal.pmed.1002335)
Supplement: S1 Table — (DOCX) [file pmed.1002335.s007.docx]

**S1 Table: Study-specific definitions of menopause**

| **Study/Country** | **Post-menopausal definition** | **Menopausal Status** | | | |
| --- | --- | --- | --- | --- | --- |
|  |  | Pre | Post | Peri* | Missing |
| Australia | Periods stopped for ≥12 months, or aged over 49.4 years at mammography if missing | 135 | 584 | 0 | 0 |
| Canada | Periods stopped for one year | 179 | 200 | 0 | 0 |
| Chile | No periods within the last year | 193 | 0 | Excluded | 0 |
| Egypt | Periods have stopped or age >55years | 244 | 250 | 0 | 0 |
| Hong Kong, China | Self-reported post-menopausal | 114 | 68 | 26 | 8 |
| India | Periods stopped permanently | 41 | 141 | 0 | 4 |
| Iran | No menstrual periods during the past 12 months | 200 | 200 | Excluded | 0 |
| Israel | No longer menstruating & >50years | 387 | 394 | 0 | 0 |
| Japan | Periods have stopped for >6 months | 200 | 190 | 0 | 0 |
| Kenya | No current periods | 165 | 187 | 0 | 0 |
| Korea | Last menstrual period >1 year | 202 | 187 | Excluded | 0 |
| Malaysia | Periods have stopped (n=144) or stopped for >6 months (n=723) | 414 | 434 | 19 | 0 |
| Mexico | Derived - Self-reported menopause, Age>51 or oophorectomy | 200 | 200 | Excluded | 0 |
| Netherlands | No periods for at least 12 months | 186 | 200 | Excluded | 0 |
| Norway | ≥6 months since last menstrual bleeding | 0 | 200 | 0 | 0 |
| Poland | No longer has periods | 200 | 198 | Excluded | 0 |
| Singapore | Stopped having menstrual periods for ≥6 months | 61 | 538 | 0 | 0 |
| South Africa | No longer has periods | 122 | 276 | 0 | 0 |
| Spain | No periods within last 12 months | 191 | 399 | 209 | 0 |
| Turkey | No periods within last year | 199 | 199 | 0 | 0 |
| UK – Ethnicity | No periods for >1 year | 66 | 445 | 59 | 12 |
| UK - MOG | Not currently menstruating | 123 | 42 | Excluded | 1 |
| UK – Da Costa | Natural (cessation of menses for at least 12 months) or surgical | 69 | 200 | Excluded | 0 |
| US - MEC Hawaii | Self-reported menopausal status | 95 | 448 | Excluded | 1 |
| US - Mayo | No menstrual periods for ≥12 months | 199 | 200 | 0 | 0 |
| US – Nurses’ Health study I and II | Derived: 1) no periods within the 12months if natural menopause, 2) bilateral oophorectomy, or 3) hysterectomy with 1 or 2 ovaries retained & ≥54 years if a smoker or ≥56 years if a non-smoker | 200 | 200 | Excluded | 0 |
| US- USC | Derived: 1) natural menopause ≥ 3 months, 2) bilateral oophorectomy, 3) age 50 years or older and on hormone therapy, 4) hysterectomy and 60 years or older at the time of mammogram, or 5) periods had stopped due to chemotherapy | 182 | 262 | 0 | 0 |
| **Total** |  | **4,567** | **6,842** | **313** | **25** |
| *Excluded: Perimenopausal women were excluded at sample selection | | | | | |
