# Supplementary material for: Mammographic density and ageing: A collaborative pooled analysis of cross-sectional data from 22 countries worldwide
Source: PLoS Med. 2017 Jun 30;14(6):e1002335. doi: 10.1371/journal.pmed.1002335 (PMC5493289; doi:10.1371/journal.pmed.1002335)
Supplement: S2 Table — (DOCX) [file pmed.1002335.s008.docx]

**S2 Table:** Difference in square-root mammographic density measures in post compared to pre-menopausal women, and by time since menopause: overall and by subgroups†
