# Supplementary material for: Mammographic density and ageing: A collaborative pooled analysis of cross-sectional data from 22 countries worldwide
Source: PLoS Med. 2017 Jun 30;14(6):e1002335. doi: 10.1371/journal.pmed.1002335 (PMC5493289; doi:10.1371/journal.pmed.1002335)
Supplement: S1 Text — (DOCX) [file pmed.1002335.s011.docx]

**S1 Text: List of studies included, by broad ethnic group**

(in decreasing order of breast area-for-BMI)

- **Black**
  - South Africa – Pink Drive Community Screening
  - Kenya - Aga Khan Hospital Low Cost Screening
  - UK-Eth-Black: Black women in the London Ethnicity Study
  - US-USC-Black: Black women in the US University of South California case control studies (controls only)
- **E. Mediterranean**
  - Israel-Arab : Israel National Breast Screening Program
  - Israel-Jewish: Israel National Breast Screening Program
  - Turkey: Bahcesehir Mammographic Screening Project
  - Iran: Isfahan University of Medical Sciences
  - Egypt - Women’s Health Outreach Program
- **White**
  - Canada-White: Ontario Breast Screening Program and Princess Margaret Cancer Center
  - Netherlands: Prospect-EPIC
  - Norway: Norwegian Breast Cancer Screening Program
  - Poland: Breast Cancer Risk Factors in Nurses
  - Spain: Determinants of Mammographic Density study
  - Australia-Australian, Australia-Greek, Australia-Italian: Melbourne Collaborative Cohort Study, Breast Screen Victoria
  - UK-Eth-White, UK-Age Trial-White, UK-London-White :White women in the London Ethnicity Study, Age Trial or in the Da Costa Study
  - US-MEC-White: White women in the US Multi-Ethnic Cohort study
  - US-Mayo-White: Mayo Mammography Health Study Cohort
  - US-NHS-White: Nurses’ Health Studies I & II
  - US-USC-White: University of South California case control studies
- **South Asian and Malay**
  - India: One-off community rural screen near Hyderabad
  - Malaysia-Indian in My Mammo study, Malaysia
  - Malaysia-Malay in My Mammo study, Malaysia
  - Singapore-Indian in Singapore Breast Cancer Screening Project
  - Singapore-Malay in Singapore Breast Cancer Screening Project
  - UK-Eth-South Asian: South Asian women (predominantly from India, Pakistan and Bangladesh) in the UK LondonEthnicity Study
- **Mestizo and Hawaiian**
  - Mestizo: Chile: Mothers of Growth and Obesity Chilean Cohort Study
  - Mestizo: Mexico: EsMaestras Teachers Cohort
  - US-MEC-Hawaiian: Hawaiian women in US Multi-Ethnic Cohort study
- **East Asian**
  - US-MEC-Japanese: US Multi-Ethnic Cohort study
  - Hong Kong: Hong Kong Sanatorium and Hospital and the University of Hong Kong
  - US-USC-Asian: University of South California case control studies
  - Japan: population-based BC screening program
  - Korea: ASAN Medical Center
  - Malaysia-Chinese in My Mammo study, Malaysia
  - Singapore-Chinese in Singapore Breast Cancer Screening Project
